# Concerted Nucleophilic Aromatic Substitution Reactions

**DOI:** 10.1002/anie.201902216

**Published:** 2019-09-13

**Authors:** Simon Rohrbach, Andrew J. Smith, Jia Hao Pang, Darren L. Poole, Tell Tuttle, Shunsuke Chiba, John A. Murphy

**Affiliations:** ^1^ Department of Pure and Applied Chemistry University of Strathclyde 295 Cathedral Street Glasgow G1 1XL UK; ^2^ Division of Chemistry and Biological Chemistry School of Physical and Mathematical Sciences Nanyang Technological University Singapore 637371 Singapore; ^3^ GlaxoSmithKline Medicines Research Centre Gunnels Wood Road Stevenage SG1 2NY UK

**Keywords:** concerted reactions, cS_N_Ar mechanism, Meisenheimer complex, nucleophilic aromatic substitution

## Abstract

Recent developments in experimental and computational chemistry have identified a rapidly growing class of nucleophilic aromatic substitutions that proceed by concerted (cS_N_Ar) rather than classical, two‐step, S_N_Ar mechanisms. Whereas traditional S_N_Ar reactions require substantial activation of the aromatic ring by electron‐withdrawing substituents, such activating groups are not mandatory in the concerted pathways.

## Graphical abstract

**Concerted or stepwise?** A class of nucleophilic aromatic substitutions has been developed that proceeds by concerted (cS_N_Ar) rather than classical, two‐step, S_N_Ar mechanisms. Whereas traditional S_N_Ar reactions require substantial activation of the aromatic ring by electron‐withdrawing substituents, such activating groups are not mandatory in the concerted pathways.

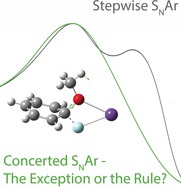

## 1 Aromatic Substitution Reactions

Substitution reactions on aromatic rings are central to organic chemistry. Besides the commonly encountered electrophilic aromatic substitution,^1^ other mechanisms include S_N_Ar nucleophilic aromatic substitutions^2^, ^3^ and the distinct but related S_N_ArH and vicarious nucleophilic substitutions,^4^ substitutions brought about through benzyne intermediates,^5^, ^6^ radical mechanisms including electron transfer‐based S_RN_1 reactions^7^ and base‐promoted homolytic aromatic substitution (BHAS) couplings,^8^ sigmatropic rearrangements,^9^ substitutions arising from deprotonation of arenes (directed metalations),^10^ the vast array of organometallic mechanisms^11^, ^12^ and S_N_1 reactions.^13^ All of these areas of chemistry are too vast to reference comprehensively, and so are simply represented here by one or two key reviews or recent references. Among these various reaction types, S_N_Ar reactions have attracted a lot of recent attention, because of a recognition that many such reactions may proceed by concerted (cS_N_Ar),^14^, ^15^ rather than classical two‐step mechanisms.

### 1.1 Classical Nucleophilic Aromatic Substitution

**Scheme 1:**
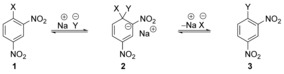
Classical two‐step mechanism for S_N_Ar reactions.

Nucleophilic aromatic substitutions have been studied at least since the 1870s.^16^, ^17^, ^18^ The long‐accepted mechanism,^4^, ^5^ exemplified in Scheme 1 for dinitroarene **1**, involved a two‐stage process that featured a Meisenheimer intermediate **2**. In these substitutions, the arene is significantly activated for substitution by the presence of one or more electron‐withdrawing substituents in the positions that are *ortho* or *para* to the site of substitution to provide resonance stabilisation, and with nitro as a favoured substituent. In Terrier's excellent book on S_N_Ar reactions in 2013,^3^ he wrote that “*concerted reactions are the exception rather than the rule*” and “*there is little doubt that most of the activated S_N_Ar substitutions must proceed through the early‐recognised addition‐elimination mechanism*”.

**Scheme 2:**
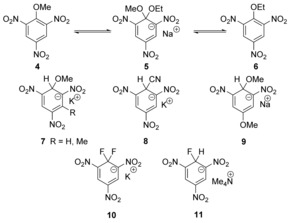
Some known Meisenheimer intermediates.

Evidence in favour of a two‐stage substitution was cited when intermediates were isolated. Thus, as reviewed by Bunnett and Zahler^2^ in 1951, a number of reactions gave rise to isolated intermediate adducts (Scheme 2). Key studies were performed by Meisenheimer,^19^ who isolated a common intermediate **5** from reaction of methyl ether **4** with NaOEt, and from reaction of NaOMe with the ethyl ether **6**. This intermediate was then decomposed into a mixture of the parent ethers on acidification. Adduct intermediates of this sort, for example, **7**–**11**, which are routinely called Meisenheimer intermediates, are widespread in organic chemistry, and are well reviewed.^20^

**Scheme 3:**
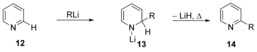
Organolithium additions to pyridine, and re‐aromatisation.

Nucleophilic aromatic substitutions are often carried out on pyridines, pyrimidines and related heterocycles, and indeed these substitutions are commonplace and important in medicinal chemistry and agrochemistry. Although intermediates from these substitutions have not been isolated where good leaving groups are present, we are familiar with isolation of intermediates where poor leaving groups are in play. Examples of intermediates at the extreme of this scale that can be isolated are the salts resulting from addition of organolithium compounds to pyridines, such as **12**, that is, compound **13** which, on heating, gives the substituted pyridine **14** with elimination of LiH (Scheme 3).^21^, ^22^, ^23^ Generation and isolation of such intermediates will be affected by the power of the ring substituents in stabilising negative charge, as well as by the p*K*_a_ values of the conjugate acids of the incoming and departing groups.

Supportive evidence in favour of the nucleophilic nature of the substitution mechanisms arises from Hammett studies, where significant positive *ρ* values are associated with the rate‐determining step. It must be remembered, when comparing *ρ* values, that they vary with the temperature of the experiments.

**Scheme 4:**
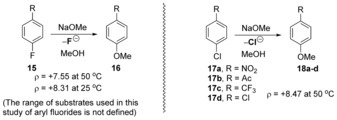
Miller's studies of Hammett correlations.

Examples reported by Miller^24^, ^25^ (Scheme 4) indicate that there is extensive negative charge build‐up in the rate‐determining step. Although the cases below in Scheme 4 have particularly high *ρ* values, it is recognised that many S_N_Ar reactions have values between +3 and +5. Looking at the substrates chosen by Miller is revealing. His series of substrates **17 a**–**d** consisted of four examples, where R=NO_2_, Ac, CF_3_ and Cl. Whereas NO_2_ and Ac are substituents that can delocalise a negative charge by resonance, clearly CF_3_ and Cl cannot, although they can contribute inductive stabilisation to different extents. Miller's Hammett analysis showed^25^ that the four substrates had an excellent correlation with σ* for these substituents,^26^ suggesting a common mechanism for them.

### 1.2 Concerted Nucleophilic Aromatic Substitution (cSNAr)—Early Developments

Although the literature adopted two‐stage S_N_Ar reactions as the norm, despite the reactivity of substrates like **17 d** studied by Miller, noted above, further anomalies began to appear. These studies have culminated in the recent paper by Jacobsen et al.,^14^ which transforms our perception of the prevalence of cS_N_Ar reactions. This will be discussed later in Section 13 of this review. Papers referenced below are cited for their relevance to cS_N_Ar reactions.

**Scheme 5:**
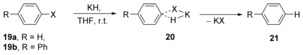
Proposal for concerted S_N_Ar reactions by Pierre et al.^27^

A very early example was the work of Pierre et al.^27^ who, in just a single paper that was published in 1980, studied the reaction of KH with aryl halides. This report simply involved hydrodehalogenation of substrates **19** in tetrahydrofuran (THF) as solvent (Scheme 5). The reactions were not pursued with detailed mechanistic investigations, but the observations made were illuminating.

By conducting the experiments with KH in [D_8_]THF, Pierre et al. were able to show that the substituting hydrogen indeed came from KH. They were able to dismiss any idea of a benzyne mechanism, since no H_2_ was evolved. The order of reactivity was: ArI > ArBr > ArCl > ArF, which is the reverse of the order often found in classical S_N_Ar reactions. Since the reactions proceeded in the absence of activating substituents like nitro groups on the ring undergoing substitution, they proposed a concerted reaction mechanism with a four‐centred transition state but, at that time, no computational methods were available to support these ideas. Perhaps because this reaction seemed so anomalous, but most likely because it was both a single paper in this area by the authors and also was not written in English, the paper received very little attention. Nevertheless, it heralded a lot of subsequent developments. We will return to this example later in this review.

**Scheme 6:**
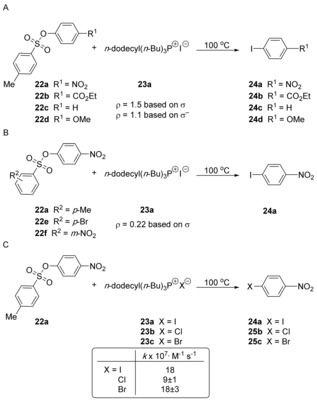
Some S_N_Ar reactions provided Hammett correlations with low *ρ*‐values.

An early study pointing to concerted nucleophilic substitution was conducted by Fry and Pienta who, in 1985,^28^ provided mechanistic evidence through Hammett correlations. When studying rate constants for nucleophilic aromatic substitution of arenesulfonate groups in **22** by halide anions in dodecyltributylphosphonium salts **23** (Scheme 6), using a range of different R^1^ substituents, Hammett plots gave reasonable fits to straight lines, with *ρ* values of +1.5 and +1.1 for σ and σ^−^ respectively (Scheme 6 A). The effect of the R^1^‐group on the rate of the reactions was therefore substantially lower than for many literature S_N_Ar reactions. Indeed, the substrates that were trialled included **22 d** (R^1^=OMe), which can clearly not provide credible stabilisation for a developing negative charge on the ring in a Meisenheimer intermediate. Importantly, the reaction series also showed some sensitivity of the transition state to the R^2^‐substituent on the leaving group (Scheme 6 B, *ρ*=+0.22). The similarity of rates regardless of the halide identity (Scheme 6 C) ruled out an S_RN_1 mechanism, as the differences in halide redox properties would require a much more substantial rate difference between the different halides. However, in their conclusion, the authors postponed speculation on the precise mechanism of their reactions.

**Scheme 7:**
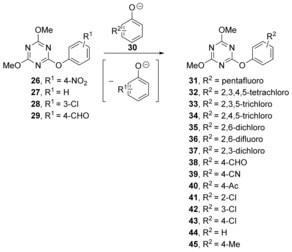
Substitutions of aryloxy‐substituted triazines.

On the other hand, Williams et al. reported a number of nucleophilic aromatic substitution reactions with concerted mechanisms on substituted 1,3,5‐triazines **26**–**29**.^29^, ^30^, ^31^, ^32^ They found that the reaction of various phenolate ions with **26** (Scheme 7),^29^, ^30^ followed a linear relationship on a Brønsted plot over a range of p*K*_ArOH_ values above and below that of the conjugate acid of the leaving group (4‐nitrophenol). The lack of curvature in the free energy relationship suggested that there was no change in mechanism when moving from strongly electron‐withdrawing groups to weakly electron‐donating groups, which is consistent with a concerted mechanism.

The same 1,3,5‐triazine core, with aryloxy and pyridine leaving groups, was also studied in aminolysis reactions with various amines.^29^, ^30^ Hammett plots for the reaction of morpholine (*ρ*=+1.65) and *N*,*N*‐dimethylaminopyridine (*ρ*=+0.82) were recorded. Detailed arguments allowed the authors to conclude that a concerted substitution was occurring. These rigorous papers were important in raising awareness of concerted nucleophilic aromatic displacements.

## 2 Some Contributions by Computational Studies

**Scheme 8:**
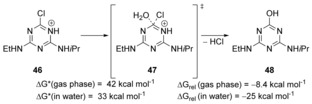
Hydrolysis of protonated triazines.

Related to these studies, computational methods were employed^33^ to examine the hydrolysis of protonated chlorotriazines for example, **46**, (Scheme 8) which are of interest in agrochemistry. In both gas phase and in water, Meisenheimer intermediates could not be located, suggesting that these reactions instead proceed in a concerted manner, albeit with high kinetic barriers, at least when a neutral water molecule was the nucleophile.

In fact, computational studies played a significant part in providing credibility for the concerted nature of cS_N_Ar reactions over the past 30 years. In all the computational studies cited here, the geometries were optimised with density functional theory (DFT) methods unless otherwise stated. We now cluster some of the computational results that suggested the cS_N_Ar mechanism, although further cases will also be referenced at appropriate places later in this review.

**Scheme 9:**
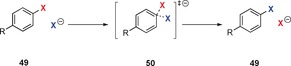
Computational investigations of identity substitutions.

Nucleophilic aromatic halogen identity‐substitution reactions were investigated computationally in the gas phase (Scheme 9) by Glukhovtsev et al.^34^ The exchange reactions of **49** with the corresponding halide anion X^−^ (for Cl, Br, I) all proceed via a Meisenheimer‐like transition state structure **50**. No intermediate was found. However, the authors observed Meisenheimer intermediates for the fluoride addition to fluorobenzene and for the chloride addition to 2,4‐dinitrochlorobenzene and picryl chloride (2,4,6‐trinitrochlorobenzene). This study was expanded by Uggerud et al.^35^ with second‐row (NH_2_^−^, OH^−^, F^−^), third‐row (PH_2_^−^, SH^−^ Cl^−^) and fourth‐row (AsH_2_^−^, SeH^−^, Br^−^) nucleophiles. Additionally, a more diverse array of substituents, R, was considered. A Meisenheimer intermediate was observed for all three second‐row nucleophiles with substituents as different as ‐NH_2_ and ‐NO_2_ (for both NH_2_^−^ and F^−^ as the nucleophile) and for substituents ‐H and ‐NO_2_ with OH^−^ as the nucleophile. For the third‐ and fourth‐row nucleophiles, concerted mechanisms were calculated in several instances. In general, a concerted mechanism was predicted for more electron‐rich aromatic systems. A stepwise mechanism with a Meisenheimer intermediate would become more favourable as electron‐withdrawing groups are attached to the aromatic ring.

Building on the halogen‐exchange reactions mentioned above, fluorodechlorination reactions and fluorodenitration reactions of aryl chlorides and nitroaryls in dimethyl sulfoxide (DMSO), were reported by Sun and DiMagno.^36^ Computational studies were performed for the fluorodenitration reactions. *para*‐Substituted nitroaryls were analysed and grouped according to the Hammett parameter of the substituents. It was observed that for substituents with a Hammett constant σ^−^ ≤0 (H and more electron‐donating substituents), the reaction proceeds via a concerted mechanism with a Meisenheimer‐like transition state.

**Scheme 10:**
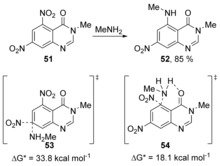
Concerted substitutions in 5,7‐dinitroquinazolin‐4‐one **51**.

Nucleophilic displacement of nitro groups, in 5,7‐dinitroquinazoline‐4‐one **51**, by methylamine as nucleophile, was reported by Goel et al.^37^ Their computational study built upon a previous experimental study^38^ that had shown that the nitro group in the *peri*‐position to the carbonyl was regioselectively displaced over the nitro group in the *para*‐position, affording **52** in 85 % yield (Scheme 10). In that experimental paper, the authors had proposed the reaction to occur via a σ‐intermediate, but evidence for this complex was not presented. Goel et al. studied the formation of the σ‐complex, but no stable complex could be found by DFT calculations.^37^ The activation energy for a concerted nitro group substitution was found to be 33.8 and 18.1 kcal mol^−1^ for *para*‐ and *peri*‐substitution via transition states **53** and **54**, respectively. The reason for the regioselectivity is given by the hydrogen‐bonding stabilisation between the amine and the carbonyl in the transition state for *peri*‐substitution, which is strong enough to divert the methylamine away from the less sterically hindered *para*‐position.

**Scheme 11:**
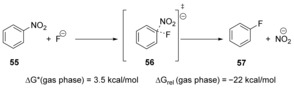
Low activation energy predicted in gas‐phase substitution.

The effect of the *medium* on substitution reactions has also been investigated widely for S_N_Ar reactions. The displacement reaction (Scheme 11) of the nitro group from nitrobenzene **55** with fluoride in the gas phase has been studied experimentally and computationally in the gas‐phase by Riveros et al.^39^ The DFT model predicts that the reaction follows a concerted pathway with a very low activation energy.

The effect of explicit solvation and counter‐cations on the displacement of a nitro group in nitrobenzene by a fluoride anion has been reported by Park and Lee^40^ through a computational approach. Including explicit solvation (two molecules of water) and different counter‐cations led to the same concerted mechanism as predicted by Riveros et al.^39^ for the gas phase, as discussed above.

The regiochemistry of displacement of halide leaving groups from poly‐halogenated substrates has been widely studied by computational methods. In 1999, Tanaka et al. reported their studies^41^ on the regiochemistry of substitution of pentafluoronitrobenzene with ammonia as nucleophile, as the solvent changed from hexane to nitromethane. These studies predicted (and provided a mechanistic proposal to explain) concerted substitution in the *para*‐position, but two‐step substitution in the *ortho*‐position.

In subsequent years, computation‐based studies on regioselectivity have been widely undertaken. Perfluoroarenes, and perhaloarenes more generally, have been the subject of a number of studies of selective substitution reactions, representing their importance in materials chemistry and in ligand generation as well as in detoxification programmes. Experimental and computational approaches have been combined by Paleta et al. in their study of pentafluorobiphenyl.^42^ With a range of N‐, O‐ and S‐nucleophiles, the regioselectivity of substitution of 2,3,4,5,6‐pentafluorobiphenyl was explored and showed significant regioselectivity for substitution of the fluorine that was *para*‐ to the phenyl group. The computational studies which used the nucleophiles i) ammonia, ii) solvated lithium fluoride [as LiF.(Me_2_O)_2_] and iii) solvated lithium hydroxide [as LiOH.(Me_2_O)_2_], mirrored the experimentally observed regioselectivity but showed that, in all cases, a concerted one‐step displacement reaction was occurring.

**Scheme 12:**
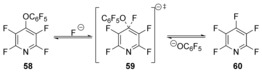
Concerted mechanism proposed in displacements of 4‐pentafluorophenoxides.

In a combined computational and experimental study, the substitution reactions of pentafluoropyridine by phenolates evidenced predominant displacement of the 4‐substituent on the pyridine.^43^, ^44^ For the resulting phenoxypyridines, extensive experimental analysis led the authors to understand that the displacement of 4‐pentafluorophenoxide (as opposed to other leaving groups) by fluoride anion from **58** (Scheme 12) was anomalous, and semi‐empirical computational studies (PM3) supported a concerted mechanism.

**Scheme 13:**
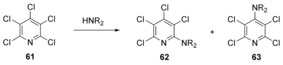
No Meisenheimer intermediates were found in computational studies on displacements on pentachloropyridine.

Following an earlier model for determining the site of substitution in aromatic perfluorocarbons,^45^ predictions of the regioselectivity of S_N_Ar reactions were made by Brinck and an AstraZeneca team including Svensson, Liljenberg et al.^46^, ^47^ based on the relative stability of Meisenheimer intermediates. As such, their model addressed the classical two‐stage mechanism. However, their computational studies could not locate these intermediates in cases where the leaving group was chloride or bromide (such as in **61**, Scheme 13), suggesting concerted reaction mechanisms in those cases.

**Scheme 14:**
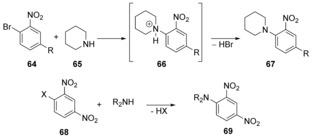
Concerted substitution reactions studied by Stenlid and Brinck.^48^

A descriptor‐based model to predict relative reactivity and regioselectivity in S_N_Ar reactions was introduced by Stenlid and Brinck.^48^ In contrast to the selectivity models presented above, this descriptor solely relies on the ground‐state electronic structure of the aromatic substrate. Consequently, it can also be applied to S_N_Ar reactions that do not proceed by a stepwise mechanism via a Meisenheimer intermediate, such as the reaction between **64** and piperidine (**65**) (Scheme 14). The series spanned examples from R=NH_2_ to R=NO_2_. The rate constants for all these examples had been reported previously. A satisfactory correlation between these constants and the newly introduced descriptor was found. The observation^48^ that, according to the computational model, reactions of **68** with secondary amines do proceed via a concerted S_N_Ar reaction was related to an extensive experimental study of 1‐X‐2,4‐dinitrobenzene with a series of secondary amines.^49^

**Scheme 15:**
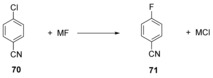
Studies on the effect of ion‐pairing, explicit hydration and solvent polarity on the fluorodechlorination reaction.

Pliego and Piló‐Veloso^50^ investigated the effect of ion‐pairing, explicit hydration and solvent polarity on the fluorodechlorination reaction of 4‐chlorobenzonitrile (**70**) (Scheme 15). This computational model predicts the reaction to follow a concerted mechanism. By varying the solvent polarity, it was found that for a given fluoride salt MF, there is a solvent with ideal polarity which just allows for the dissociation of the ion pair but does not solvate the fluoride ion too strongly.

**Scheme 16:**
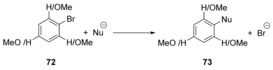
Substitution reactions of bromomethoxybenzenes.

In a more recent contribution, Silva and Pliego investigated^51^ S_N_Ar reactions on bromobenzene and *(ortho*‐, *meta*‐, or *para*‐) methoxybromobenzenes with different nucleophiles in the gas phase and in solution phase by computational methods (Scheme 16). A concerted mechanism was observed with hydroxide, cyanide, and methoxide nucleophiles attacking bromobenzene in the gas phase (albeit the transition state energy for the reaction with cyanide was high (ΔG*=27.2 kcal mol^−1^). Including solvent effects in their computations made all three reactions kinetically less favourable (e.g. hydroxide in DMSO: ΔG*=29.3 kcal mol^−1^; in MeOH: ΔG*=37.8 kcal mol^−1^. These barriers are markedly higher than in the gas phase ΔG*=1.6 kcal mol^−1^).

No change in mechanism is mentioned when going from gas‐phase to solution‐phase models. Interestingly, when the authors investigated *meta*‐methoxybromobenzene with hydroxide, methoxide and cyanide as the nucleophile, they obtained lower activation barriers (e.g. ΔG*=25.8 kcal mol^−1^ for *m*‐methoxybromobenzene with methoxide in DMSO vs. ΔG*=27.1 kcal mol^−1^ for bromobenzene with the same nucleophile in the same solvent).

**Scheme 17:**
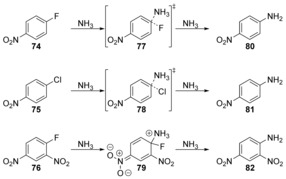
Gas‐phase reactivity with ammonia as nucleophile.

**Scheme 18:**
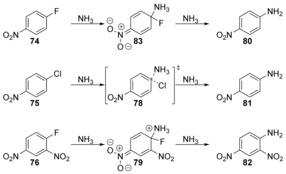
Reactivity in solution with ammonia as nucleophile.

The effects of solvation on S_N_Ar reactions in liquid ammonia and in the gas phase by a combination of metadynamics and committor analysis methods have been studied by Moors et al.^52^ They found that for 4‐nitrofluorobenzene (**74**), the reaction proceeded via a concerted mechanism in the gas phase via transition state **77**, but via an intermediate Meisenheimer complex **83** in solution (Schemes 17 and 18). For 4‐nitrochlorobenzene (**75**), the reaction proceeded via a concerted mechanism via transition state **78** in both the gas phase and in solution, and for 2,4‐dinitrofluorobenzene (**76**), the reaction occurs via a Meisenheimer intermediate **79** in both solution and the gas phase.

## 3 Fluorodeoxygenation of Phenols and Derivatives

**Scheme 19:**
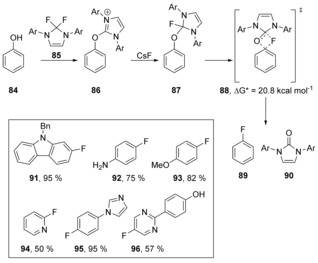
Ritter's studies^53^, ^54^, ^55^, ^56^ on the deoxyfluorination of phenols.

Fluoride played a major role in the important studies^53^, ^54^, ^55^, ^56^ by Ritter et al. who had already reported^53^ the deoxyfluorination reaction of phenols **84** with PhenoFluor **85** (Scheme 19).^54^ Intermediate **87** (Ar=Ph) was independently synthesised and treated under the reaction conditions, and afforded the corresponding aryl fluoride **89**.

When DFT studies were carried out, a single transition state, **88**, was observed, which is characteristic of a concerted mechanism. A large primary ^16^O/^18^O kinetic isotope effect (KIE) (KIE=1.08±0.02) was observed, showing that the cleavage of the C−O bond is involved in the rate‐determining step. A Hammett plot also shows that there is no change in mechanism when moving from electron‐deficient phenols to electron‐rich phenols (*ρ*=+1.8) indicating that there is not a build‐up of full negative charge in the ring at the transition state.^56^ The formation of the urea by‐product is also highly exergonic, which contributes to the driving force for this reaction. These reactions feature *spiro* transition states, further examples of which will appear later in this review (Sections 7 and 8).

**Scheme 20:**
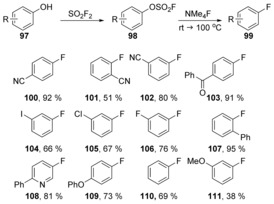
Examples of deoxyfluorination by Sanford et al.

On a related theme, Sanford et al. reported a mild deoxyfluorination of phenols **97** via aryl fluorosulfonate intermediates **98**.^57^ This transformation was found to be compatible with *ortho*‐, *meta*‐, *or para*‐electron‐withdrawing groups, and could also be applied to electron‐neutral and moderately electron‐rich substrates to provide fluorinated products **100**–**111** (Scheme 20).

**Figure 1:**
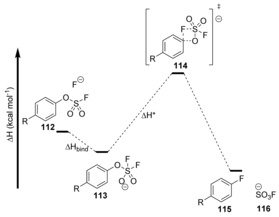
Energy profile for conversion of **112** to fluoroarene **115**.

Computational data suggest that the binding of fluoride in **112** to sulfur to form pentacoordinate sulfonate **113** is enthalpically favourable and the activation enthalpy to the transition state (ΔH*) was found to be feasible at room temperature (Table 1). The transition state **114** was shown to involve concerted formation of the C−F bond and cleavage of the C−O bond without the formation of a Meisenheimer intermediate (Figure 1).

**Table 1:** Enthalpy changes associated with Figure 1

Entry	R (structure **112**)	Δ*H*_bind_ [kcal mol^−1^]	ΔH* [kcal mol^−1^]
1	CN	−4.1	13.2
2	CF_3_	−3.5	15.6
3	H	−1.7	20.8
4	Me	−1.3	22.2
5	OMe	−1.1	24.0

## 4 Aminodeoxygenation of Phenol Derivatives

**Scheme 21:**
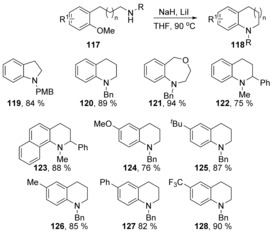
Intramolecular nucleophilic amination of methoxyarenes.

**Scheme 22:**
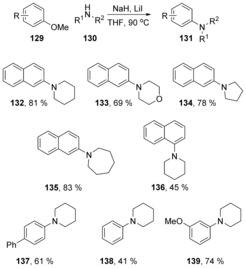
Intermolecular nucleophilic amination of methoxyarenes.

Chiba et al. have recently reported remarkable reactions of a sodium hydride–lithium iodide composite. One of the reaction types reported by that team promoted the nucleophilic amination of methoxyarenes **117**, via intra‐ (Scheme 21) and intermolecular (Scheme 22) reactions.^58^ This methodology was compatible with electron‐donating and electron‐withdrawing substituents on the methoxyarene.

**Figure 2:**
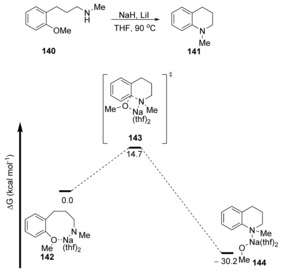
Free energy profile for the cyclisation of amide salt **142**.

A Hammett plot with *p*‐substituted methoxyarenes **129** showed that the *ρ*‐value was low (*ρ*=+1.99). The proposal of a concerted mechanism was backed up by computational analysis, where a single transition state was observed for the conversion of **140→141** with formation of a partial negative charge, consistent with a cS_N_Ar process (ΔG*=14.7 kcal mol^−1^, Figure 2). Chiba's demethoxylation studies feature deprotonated amines as nucleophiles. Demethoxylation by a hydroxycyclopentadienyl iridium hydride nucleophile has been proposed as a concerted nucleophilic aromatic substitution by Kusumoto and Nozaki,^59^ although no mechanistic evidence has yet been revealed to support this.

**Scheme 23:**
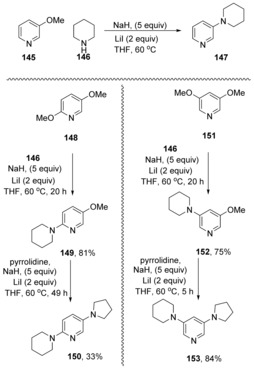
Sequential substitutions of methoxyarenes by amines.

Chiba and co‐workers extended their chemistry with NaH and additive salts to perform further intermolecular displacements.^60^ For example, substitution of the methoxy group in 3‐methoxypyridine (**145**), (Scheme 23) by piperidine **146** was achieved in high yield using sodium hydride with LiI as additive. With NaI as alternative additive, the reaction proceeded in much poorer yields and with NaH alone, no reaction was seen.

This reaction is quite flexible. In the dimethoxy case **148**, substitution at the 2‐position occurs first to give **149**, but the product can undergo a second substitution by a different amine to give **150**. In 3,5‐dimethoxy case **151**, iterative diamination can again be achieved. In these cases, no Hammett correlations have been published, but the displacement from the unactivated 3‐position of a pyridine identifies these reactions as prime candidates for cS_N_Ar pathways.

## 5 Hydrides as Nucleophiles

**Scheme 24:**
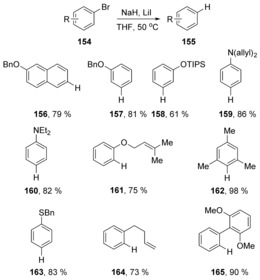
Hydrodehalogenations effected by sodium hydride‐lithium iodide complex.

Recent discoveries relating to concerted aromatic substitutions have seen several that feature hydride as nucleophile or base. Chiba et al. recently reported the hydrodehalogenation of haloarenes **154**, (Scheme 24) by their sodium hydride–iodide composite.^61^ Without the addition of the iodide salt, sodium hydride cannot carry out this special function.

**Figure 3:**
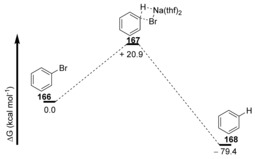
Free Energy profile for reaction of bromobenzene with solvated monomeric sodium hydride.

Various aryl bromides were reduced under these conditions, with both electron‐rich and electron‐deficient substituents being equally tolerated. Computational studies show a highly exothermic reaction with a single transition state **167** for concerted nucleophilic aromatic substitution, with an energy barrier of 20.9 kcal mol^−1^ (Figure 3). The Hammett plot using NaH with NaI, converting iodoarenes to arenes in THF at 85 °C shows a linear correlation with *ρ*=+0.47, which is supportive of a cS_N_Ar process.

As the cS_N_Ar pathway is initiated by an interaction between the hydride donor and the π* orbital of the aromatic ring, it was reasoned that this methodology could also be applicable to the reduction of haloalkenes, upon treatment with the sodium hydride‐iodide composite.

**Scheme 25:**
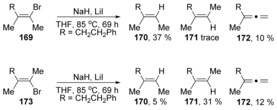
Stereo‐retention prevails in hydrodehalogenations of vinyl bromides.

This was indeed the case, with retention of configuration being observed as the major product for both (*Z*)‐ and (*E*)‐ alkenes **169** and **173** (Scheme 25).^61^, ^62^

**Scheme 26:**
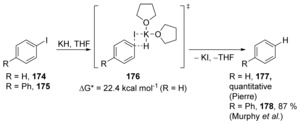
Support for four‐centred transition state in the Pierre reaction.

Murphy, Tuttle et al. recently reported on the solvent‐dependent role of potassium hydride in haloarene reduction.^63^ Pierre et al. had proposed a cS_N_Ar mechanism for dehalogenation of haloarenes in 1980, as mentioned earlier.^27^ They had verified that the hydrogen atom delivered to the aryl halide had come from KH. They had ruled out a benzyne intermediate in their reactions, and presented their proposal based on the observed order of reactivity (ArI > ArBr > ArCl) which was in contrast to the normal order of reactivity in a standard S_N_Ar reaction on iodobenzene **174** (Scheme 26). Pierre's proposal was therefore revolutionary, being made before computational methods became widely available. Murphy and Tuttle's investigation confirmed Pierre's proposed mechanism computationally, with a Gibbs free energy barrier of 22.4 kcal mol^−1^. Studies carried out in [D_8_]THF also reveal that the H‐atom in **178** comes from the KH rather than from the solvent, in line with Pierre's claim. Surprisingly, in benzene as solvent, Murphy and Tuttle showed that a quite different electron transfer mechanism played an important role in reduction of haloarenes with KH.

**Scheme 27:**
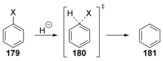
Concerted displacements of halides from haloarenes by naked hydride ions are predicted from computation.

Computational studies on hydrodehalogenation of haloarenes by cS_N_Ar have been reported by Cramer et al.^64^, ^65^ In all cases, the transition state for the addition of hydride to a substituted site led to concerted displacement of the halide anion via transition state **180** (Scheme 27).

**Scheme 28:**
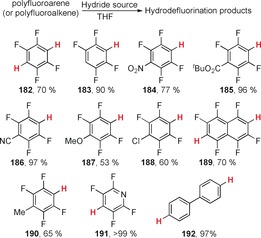
Products of hydrodefluorination from the study of Ogoshi et al. **H** indicates an H atom that has displaced F; only the major product isomer is shown in each case.

**Scheme 29:**
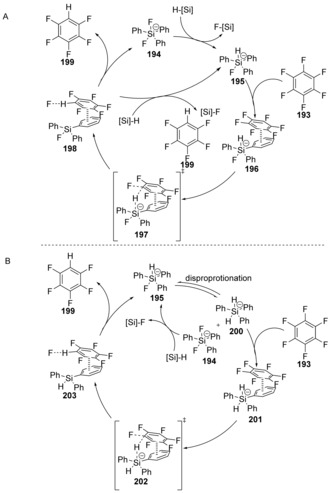
Proposed cycles for hydrodefluorination by TBAT (**194**) and a silane.

Ogoshi et al. recently reported^66^ a catalytic and regioselective hydrodefluorination of polyfluoroarenes (Scheme 28) and polyfluoroalkenes using silanes (Ph_3_SiH, MePh_2_SiH, Me_2_PhSiH or Et_2_SiH_2_) and catalytic tetrabutylammonium difluorotriphenylsilicate (TBAT **194**, Scheme 29).

This hydrodefluorination process tolerated many other functional groups, including esters, nitriles and nitro groups. Two mechanistic cycles were proposed by the group, based on DFT studies, both of which proceed through a cS_N_Ar displacement step.

The first cycle (Scheme 29 A) involves generation of **195** from TBAT (**194**) and the hydrosilane. This can then coordinate to the polyfluoroarene **193** via π–π stacking, affording **196**. The cS_N_Ar step can then occur with hydride from the silicate displacing a fluoride in the transition state **197**. The eliminated fluoride can then either be trapped intramolecularly by a fluorosilane or intermolecularly by a hydrosilane to regenerate **194** or **195**, respectively. The alternative mechanistic cycle involves dihydrosilicate **200** as an intermediate, which can be formed by disproportionation of **195** (Scheme 29 B). Hydride transfer within complex **202**, displaces a fluoride ion from the polyfluoroarene. The displaced fluoride can then be trapped by the hydrosilane to regenerate **195**. Computational data for the mechanisms in Scheme 29 show that the Gibbs free energy barriers for the key substitution steps are 19.0 kcal mol^−1^ and 10.8 kcal mol^−1^, respectively. Meisenheimer intermediates were not detected for either pathway, indicative of cS_N_Ar reactivity.

## 6 P, N, Si, C Nucleophiles

**Scheme 30:**
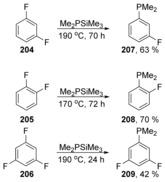
Phosphinodefluorination of aryl fluorides.

The range of nucleophiles was further widened when Würthwein et al. reported the reaction of di‐ and trifluorobenzenes **204**–**206** with Me_2_EM (E=P, N; M=SiMe_3_, SnMe_3_ Li) (Scheme 30).^67^, ^68^ To illustrate the utility of the transformation, phosphane products were later used as ligands in polyfluorophosphane palladium dichloride complexes. Computational chemistry was again shown to be a useful tool in predicting the mechanism of this reaction, indicating a single transition state with no Meisenheimer adduct formation, that is, a cS_N_Ar mechanism.

**Figure 4:**
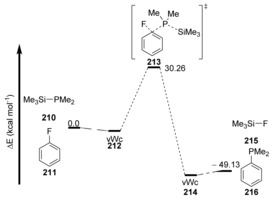
Energy profile of phosphinodefluorination reaction.

The single transition state corresponds to the simultaneous C−E bond formation, and in the case of Me_2_PSiMe_3_ with fluorobenzene, provided a barrier of ΔE*=30.3 kcal mol^−1^ for the formation of the C−P bond and the Si‐assisted loss of fluoride (Figure 4). Di‐ and trifluorobenzenes were also examined experimentally and by computation, and provided faster reactions and lower calculated barriers. Compounds **210** and **211** form a van der Waals complex (vWc) **212**, followed by a cS_N_Ar reaction through **213**, forming van der Waals complex **214**. Dissociation of this complex affords products **215** and **216** (Figure 4).

**Scheme 31:**
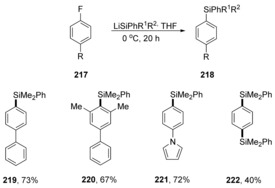
Products arising from silyldefluorination reactions.

Aryl fluorides have recently been subjected to quite a different cS_N_Ar reaction by Würthwein, Studer et al., using silyllithium reagents as nucleophiles.^69^ This was an interesting development, as previous reactions of aryl halides (notably iodides) with similar reagents had led to substitution directly on the halogen atom to give a silyl halide and an aryllithium as a reactive intermediate that then conducted an S_N_2 reaction on the silyl halide. In this case, however, aryl fluorides underwent cS_N_Ar. Hammett studies gave a *ρ* value of +3.2, and computational investigation afforded Gibbs free energies of activation of 19–21 kcal mol^−1^ (Scheme 31). Very recently, two related studies have appeared from other groups.^70, 71^

**Scheme 32:**
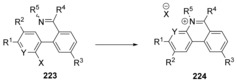
Hartley's cS_N_Ar route to phenanthridinium salts.

A flexible route to phenanthridinium cations **224** was published by Hartley et al. using an imine nucleophile to displace a halide (Scheme 32).^72^ The imine **223** which is formed in situ is not isolated, but directly converted to product **224** by heating. The mechanism of the ring‐forming S_N_Ar reaction from **223** to **224** was investigated computationally with model compounds. The models were chosen to closely represent the synthesised molecules. It was found that for all examined model compounds, the reaction proceeds via a concerted S_N_Ar pathway. In particular, a concerted mechanism was not only observed for examples with electron‐donating substituents (e.g. *p*‐MeO) but also for examples with electron‐withdrawing substituents (e.g. *p*‐NO_2_). All transition states were accessible (ΔG*=between 17 kcal mol^−1^ and 28 kcal mol^−1^) with lower energy barriers for examples with more electron‐withdrawing substituents, as expected.

**Scheme 33:**
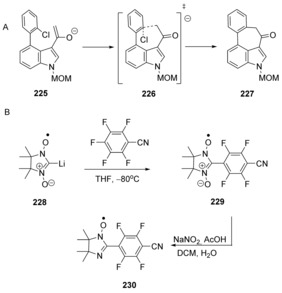
Carbon nucleophiles in cS_N_Ar displacements (DCM: dichloromethane; AcOH: acetic acid).

Carbon nucleophiles were used by Médebielle, Rossi et al. in the synthesis of tetracyclic indoles, for example, **227** (Scheme 33 A) and azaindoles.^73^ Computational studies were used to probe the mechanism of the reaction. Electron transfer was considered, but gave very high energy barriers. The most reasonable proposal was that the reactions proceeded by nucleophilic aromatic substitution. The calculated reaction profile showed no intermediates, that is, it was a cS_N_Ar reaction.

An unusual nucleophile **228** was employed by Tretyakov et al.^74^ (Scheme 33 B) to afford a new nitronyl nitroxide **229**. DFT calculations supported the observed regioselectivity and indicated that the reaction follows a concerted pathway. The zwitterionic product was treated with sodium nitrite in acetic acid to yield the nitroxyl **230**.

## 7 Organic Rearrangements via Spiro Species: Intermediates or Transition States?

**Scheme 34:**
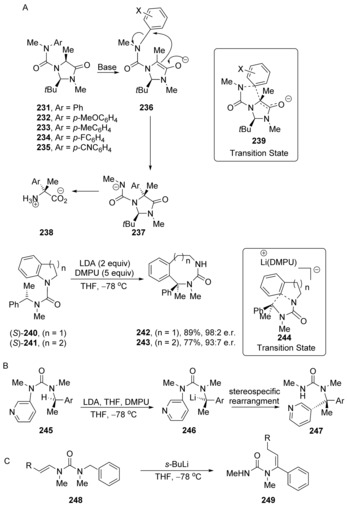
A) Stereocontrol in Clayden's aryl transfer reactions; B) earlier aryl reactions showing nucleophilic substitution at the *meta‐* position of a pyridine; C) vinyl transfer reactions.

*Spiro* transition states appeared in the fluorodeoxygenation section of this review, but *spiro* species occur much more widely, as seen in this and the following section of this review. Tell‐tale signs of concerted nucleophilic substitutions arise when the arene at which substitution is occurring has no substituents to significantly stabilise a Meisenheimer intermediate. This is the case in Clayden's stereocontrolled arylation of amino acids.^75^, ^76^ Here, infrared spectroscopy was used to follow the conversion of alanine derivatives **231**–**235**, via their enolates **236** into products **238** as shown in Scheme 34. Conformational control of anilides plays an important role here. In tertiary anilides (e.g. **231**), the aryl group is aligned strictly *anti* to the carbonyl group.^77^

Formation of enolate **236** ensues, and transfer of the aryl group then occurs with control of stereochemistry to afford the anion **237**, from which the amino acid product **238** was isolated. Plainly, without appropriate stabilising substituents on the arene, no Meisenheimer intermediate can be detected or envisaged, and yet the transformation occurs smoothly in high yield. A Hammett plot for substrates **231**–**234** revealed *ρ*=+4.5 in this case against σ^−^, indicating significant charge build‐up on the arene and showing that the arene‐transfer step is the rate‐determining step. The leaving groups in these cases are amide anions. The fact that they are not good leaving groups, is consistent with the need for significant charge build‐up on the ring in the transition state, before the departure of the leaving group is triggered.

On the other hand, for substituents on the arene where σ^−^ > +0.2, the arene transfer is facilitated and is apparently no longer rate‐determining; in those cases, the enolate formation step takes over this role.

Prior to this most recent work, Clayden et al. had studied extensive alternative applications of these aryl transfer reactions, notably in ring‐expansion reactions,^78^, ^79^, ^80^, ^81^, ^82^, ^83^ for example, with substrates **240**, **241**.^78^ Although Hammett plots are not reported for these series, the analogy to the amino acid cases just discussed make it highly likely that they follow cS_N_Ar pathways through transition states **244**. In the presence of dimethylpropyleneurea (DMPU) and lithium diisopropylamide (LDA), ring‐expanding isomerisation was effected with excellent retention of stereochemistry. Thus, the initially generated benzyllithium is configurationally stable for the period needed to carry out the rearrangement.

Scheme 34 B and 34 C show additional applications of transfer reactions. The attack of the benzyllithium nucleophile **246** on the *meta*‐position of the pyridine ring is again a strong indicator of cS_N_Ar reactivity. The stereoselectivity of the reaction is again noteworthy. Both this example and the vinyl transfer reaction with substrate **248** in Scheme 34 C bear resemblance to the results of Chiba et al. in Sections 4 and 5 of this review.

**Scheme 35:**
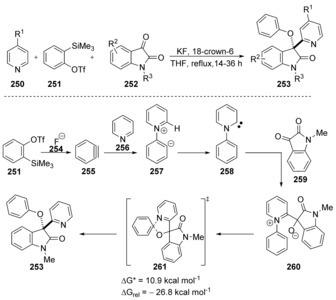
Unexpected product **253**, together with a proposal for its mechanism of formation.

The substitution steps in Clayden's work are examples of Truce‐Smiles rearrangements,^84^ intramolecular substitution reactions that go through a *spiro* transition state or intermediate, with carbon nucleophiles and nitrogen leaving groups. A quite different example of cS_N_Ar chemistry was reported by Coquerel^85^ in 2013 that also involved a Smiles‐type rearrangement, this time with an oxygen nucleophile and a nitrogen leaving group. In a study of the reactions of benzyne with pyridine, they isolated an unusual product of rearrangement, **253**. This was rationalised through the pathway shown (Scheme 35) where generation of the zwitterion **257** leads to internal deprotonation to give pyridine carbene **258**. This nucleophilic carbene then reacted with the reactive ketone carbonyl group of N‐protected isatin **259**, and the resulting alkoxide then secured a phenyl transfer reaction to liberate a neutral pyridine nitrogen in **253**.^85^ Computational studies revealed that the conversion of **260** to **253** was occurring by a concerted process. In the transition state **261**, the carbon atom undergoing substitution adopts sp^3^‐like geometry as characterised by the computed bond angles and bond lengths. The phenyl group bearing the pyridinium substituent in **261** did not feature any activating substituent [other than the leaving group] and the activation barrier (ΔE*) was very accessible at 10.9 kcal mol^−1^.

**Scheme 36:**
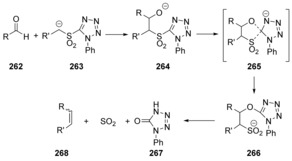
The Julia–Kocieński reaction features concerted displacement at the tetrazole ring.

The Julia‐Kocieński reaction^86^ (Scheme 36) also involves a Smiles‐type rearrangement step and has been studied in detail with computational methods. The effect of coordinating counter cations and different solvents on the *Z*/*E* selectivity of the product alkenes is rationalised. It was found that the rearrangement step through *spiro* species **265** (Scheme 36) follows a concerted mechanism in all examined cases (different solvents and counter‐ions). The authors note that at no point during this rearrangement is a significant amount of negative charge transferred onto the tetrazole ring. Instead the negative charge is directly transferred from the attacking alkoxide nucleophile to the sulfur atom of the leaving group. The transition state is asynchronous and early. The new carbon‐oxygen bond is formed to a significant extent while the carbon‐sulfur bond still is mainly intact.

As mentioned above, the Smiles rearrangement is an intramolecular substitution reaction featuring a *spiro* species on the reaction path. Concerted pathways had been considered for other examples of the Smiles rearrangement early on.^87^ In contrast to the cases just cited, computational studies showed that several examples of the reaction proceed by a stepwise mechanism via a Meisenheimer intermediate.

**Scheme 37:**
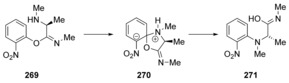
A stepwise mechanism was predicted for this Smiles rearrangement from computational studies.

The Smiles rearrangement of **269** was investigated computationally with a range of different functionals (Scheme 37).^88^ It was found that, depending on the functional, structure **270** can either be optimised as a local minimum or as a transition state. Benchmark models at Møller–Plesset MP2/6‐31+G(d,p) and MP4(SDQ)/6‐31+G(d,p) level of theory showed that **270** is an intermediate. In general, functionals with <10 % Hartree–Fock (HF) exchange were unable to correctly identify **270** as a local minimum and predicted a concerted mechanism instead. Notably, the popular B3LYP functional was found to fail to predict the correct stepwise mechanism despite having 20 % HF exchange. M06, M06‐2X and ωB97X were found to give results satisfactorily close to the Møller–Plesset results, that is, they all predicted a stepwise mechanism with reasonably accurate barrier heights.

**Scheme 38:**
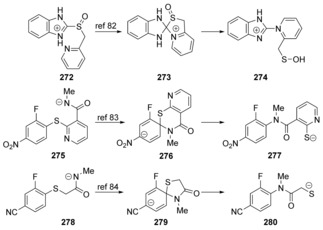
Further examples of Smiles rearrangements where computational research predicts stepwise mechanisms.

Further examples **272**,^89^ **275**,^90^ **278** 91 of Smiles rearrangements were explored (Scheme 38) through computational methods^89^, ^90^, ^91^, ^92^ and each of these gave clear intermediates, rather than concerted *ipso*‐substitution reactions. So, overall, the studies on Smiles rearrangements indicate that there is a delicate balance between concerted and stepwise substitution reactions.

## 8 Newman–Kwart and Related Rearrangements

**Scheme 39:**
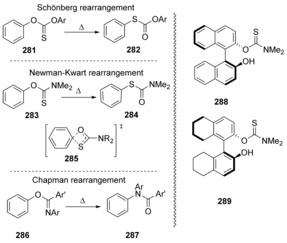
The Newman–Kwart and related rearrangement reactions.

Closely related to the above reactions that featured five‐membered ring *spiro* species, four‐centred transient *spiro* rings are proposed for a number of other rearrangement reactions, namely the Chapman, Schönberg^93^ and Newman–Kwart rearrangements. Of these, the Schönberg rearrangement of diarylthionocarbonates **281** to diarylthiolcarbonates **282** (Scheme 39) was studied intensively first.

Tarbell et al.^94^ proposed a four‐centred transition state to be at the heart of this rearrangement. The reactions are accelerated by electron‐withdrawing substituents in the aryloxy ring. The Newman–Kwart rearrangement, for example, **283→284**, was excellently reviewed in 2008 by Lloyd‐Jones et al.^95^ Relles et al. found^96^ similarities between the Chapman and Newman–Kwart rearrangements on studying their properties separately through Hammett correlations *ρ*=+1.62 for the Newman–Kwart rearrangement and +1.63 for the Chapman rearrangement. A similar assessment by Miyazaki^97^ versus σ^−^ gave *ρ*=+1.83 for the Newman–Kwart rearrangement and *ρ*=+1.06 for the Chapman rearrangement. Woodward, Lygo et al.^98^ (2003) conducted computational studies on the Newman–Kwart rearrangement of two analogous series of atropisomerically pure thionocarbamates, one derived from binol (**288**) and one from octahydrobinol (**289**) (Scheme 39). They observed experimentally that the octahydrobinol cases rearranged essentially without racemisation, while the binol case showed significant racemisation. Their computational studies at different levels of theory showed that the barrier for the rearrangement of the octahydrobinol case was notably lower than for the binol case, while the barrier for thermal racemisation of the substrates had the reverse order. Jacobsen and Donahue^99^ used DFT calculations to back the proposal for a four‐centred transition state.

More recently, a radical cation version of the Newman–Kwart rearrangement has been discovered^100^ that proceeds under mild conditions and that has quite a different response to substituents than in the thermal rearrangement. Cramer has reported recent studies that provide further computational characterisation of the thermal Newman–Kwart rearrangement as well as its radical cation counterpart; the radical cation variant is also viewed as being a concerted substitution reaction.^101^, ^102^, ^103^

## 9 Sulfur Nucleophiles

**Scheme 40:**
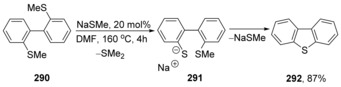
cS_N_Ar reactions in the formation of dibenzothiophenes.

Sulfur nucleophiles have also featured prominently in the recent literature. Tobisu, Chatani, et al. have just reported^104^ an unusual outcome to reaction of 2,2′‐bis(methythio)‐1,1′‐biaryls **290** (Scheme 40) with catalytic amounts of methanethiolate salts in dimethylformamide (DMF) as solvent.

Here, the reaction commences with demethylation of the ArS–Me bond to afford an arenethiolate **291**, which then attacks the adjacent arene, displacing methanethiolate anion to complete a cycle by forming **292**. Computational studies were unable to identify any intermediate in the latter step, which therefore appears to be concerted.

**Scheme 41:**
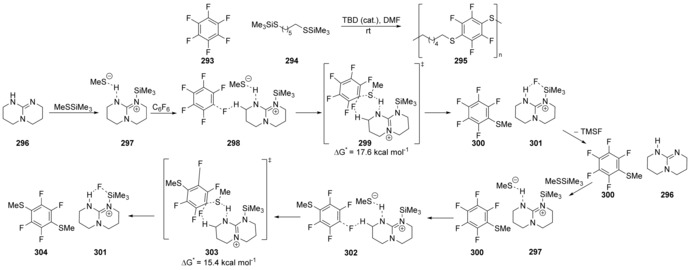
Substitution of perfluorophenylbenzenes by methanthiolate occurring through highly ordered transition states.

Hedrick, Alabugin et al. recently reported^105^ that the synthesis of fluorinated poly(arylthioethers) **295** proceeds via a concerted mechanism (Scheme 41). Through computational studies, it was shown that firstly triazabicyclodecene (TBD), **296**, nucleophilically attacks the trimethylsilyl (TMS) group of MeSSiMe_3_, displacing a methanethiolate anion which hydrogen bonds to the TBD‐TMS cation forming **297**. This then forms a complex **298** with hexafluorobenzene (**293**), before the methanethiolate anion displaces fluoride in a concerted manner in transition state **299**, aided by hydrogen bonding between the fluorine and the amine catalyst.

Dissociation of Me_3_SiF occurs from **301**, regenerating **296**, followed by complexation of another MeSSiMe_3_ and the monothiolated arene **300**, forming **302**. A second concerted displacement occurs *para* to the first displacement, due to stabilisation from the first methanethiolate group acting as a σ‐acceptor (via transition state **303**). Dissociation of fluorotrimethylsilane regenerates the catalyst **301** and affords dithiolated product **304**.

**Scheme 42:**
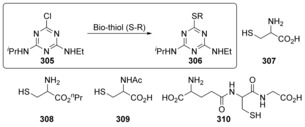
Thio‐dehalogenation of atrazine **305** occurs on the borderline between concerted and stepwise mechanisms.

Calfumán et al. carried out an experimental and computational study into the reaction of atrazine **305** with various bio‐thiols **307**–**310**, and propose that these reactions occur on the borderline between concerted and stepwise mechanisms (Scheme 42).^106^ A Brønsted plot shows *β*=+0.5, which corresponds to a stepwise mechanism via a Meisenheimer intermediate, however, computational analysis of the intrinsic reaction coordinate reveals that no Meisenheimer intermediate can be found. The authors suggest that this may be because the loss of the chloride is extremely fast.

**Scheme 43:**
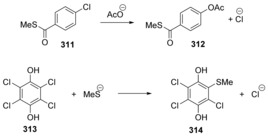
Bio‐inspired substitution reactions.

Investigations^107^ of the nucleophilic aromatic displacement of chloride from a 4‐chlorobenzoyl CoA model compound **311** (Scheme 43) with the acetate ion suggest that this reaction proceeds via a concerted mechanism. In the same study the nucleophilic aromatic substitution of chloride from tetrachlorohydroquinone **313** with thiomethanolate was found to proceed via a concerted mechanism (with the semi‐empirical method, PM3). The authors point out that in solution phase (or on the enzyme) the accumulating negative charge in the transition state may be stabilized. Consequently, the reaction that proceeds via a concerted pathway in the gas phase could proceed via a stepwise pathway with a Meisenheimer intermediate in solution phase.

## 10 Hypervalent Iodine Substrates

**Scheme 44:**
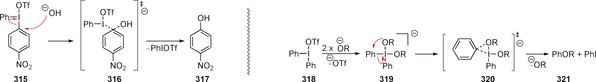
cS_N_Ar substitutions on arenes with a hypervalent iodine substituent.

Olofsson et al. investigated^108^ *O*‐arylations with diaryliodonium salts through experimental and computational methods, using hydroxide ion, alcohols and phenols as nucleophiles. The iodonium salts are represented as covalent diaryliodine(III) triflates, for example, **315** (Scheme 44) that undergo displacement of the triflate (‐OTf) leaving group in the Ar_2_I‐OTf molecule by a nucleophile, before other chemistry transpires. The overall mechanistic picture is complex in that different mechanistic possibilities arose depending on the nucleophile and the iodine(III) substrate. However, in electron‐poor iodine(III) substrates such as (*p*‐NO_2_C_6_H_4_)I(Ph)OTf, **315**, they propose a direct *ipso* displacement by hydroxide ion at the C−I bond of the nitroarene ring to lead to *p*‐nitrophenol. They similarly represent an attack of alkoxides on Ph_2_IOTf (**318**), as involving an initial conversion of the triflate complex to the dialkoxy “ate” complex that then undergoes concerted substitution at the *ipso* centre as shown. Additionally, they show oxidation of alcohols by the iodine(III) substrates as involving concerted delivery of hydride to the *ipso* carbon with loss of iodoarene.

Similar reactions were more recently carried out^109^ on cyclic secondary amines by Stuart et al., as well as primary amines,^110^ by Olofsson et al. Stuart describes the final step of his proposed reaction mechanism as a reductive elimination whereby Ar−N bonds were created in the same step as the Ar−I bond was being cleaved. No computational or Hammett or other analyses of these reactions are available at the time of writing this review, but the analogy to the reactions of Olofsson et al. with alcohols is clear.

**Scheme 45:**
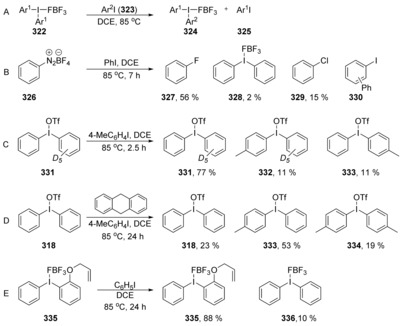
Mechanistic studies on nucleophilic aromatic substitution reactions on hypervalent iodine substrates. (DCE: 1,2‐dichloroethane).

Uchiyama et al.^111^ provided a route to *ortho*‐iodo diaryl ethers. They found that upon studying aryl‐exchange reactions of diaryl‐λ^3^‐iodanes with aryl iodides, the aryl exchange occurred via what they termed a cS_N_Ar process (Scheme 45 A), but different from those encountered so far in this review. S_N_1 reactivity, benzyne pathways, and single electron transfer were all ruled out. S_N_1 was ruled out by the absence of any fluoroarene that would be expected to form if the reaction proceeded via an aryl cation, such as seen in the formation of fluorobenzene **327** from benzenediazonium **326** (Scheme 45 B). A benzyne pathway was ruled out by deuterating one aryl group on **331** and no D/H scrambling was observed (Scheme 45 C). Aryl radical intermediates were ruled out by the addition of a radical scavenger, 9,10‐dihydroanthracene, (Scheme 45 D) and by preparation of a radical clock substrate **335**, which did not afford any cyclised products (Scheme 45 E).

**Scheme 46:**
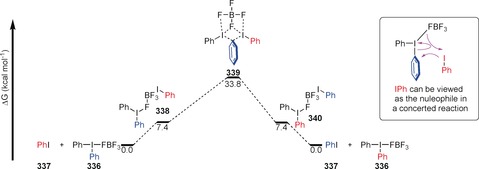
Free Energy profile for nucleophilic aromatic substitution reactions on hypervalent iodine substrates.

Kinetic data suggest that both reagents are involved in the transition state. Density Functional Theory (DFT) calculations suggest that the reactants **336** and **337** weakly coordinate through the BF_4_ ion before a concerted aryl group migration occurs via two I(II) species with some *positive* charge development at the *ipso*‐carbon (**339**, Scheme 46). Dissociation of the aryl iodide from the tetrafluoroborate affords the products **336** and **337**. The reaction is reversible, and proceeds with thermodynamic control.

**Scheme 47:**
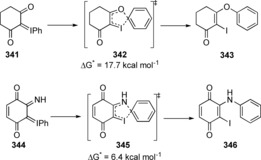
Aryl migration in iodonium ylides.

Bakalbassis et al. used computational methods to study the reaction of aryl migration in aryliodonium ylides **341** and **344**, and found this to be a concerted process with a barrier of 17.7 kcal mol^−1^ and 6.4 kcal mol^−1^ for substrates **341** and **344**, through transition states **342** and **345** respectively (Scheme 47).^112^

## 11 Reactions of Arenediazonium Salts

**Scheme 48:**
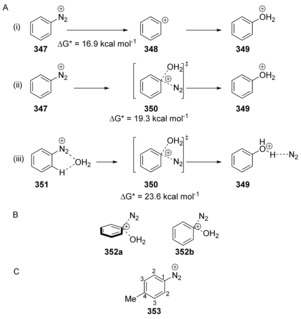
Substitution reactions of arendiazonium salts by water.

Computational studies into the reaction of the benzenediazonium ion **347** with water have been reported by Glaser et al.^113^ They considered three mechanisms (Scheme 48 A); i) a unimolecular S_N_1Ar mechanism with generation of an intermediate phenyl cation; ii) a bimolecular S_N_Ar that proceeds without the pre‐ and post‐coordination of the water and the diazonium salt; and iii) a bimolecular S_N_Ar that proceeds with pre‐ and post‐coordination of the water and diazonium salt.^114^

The authors propose that the transition state for the reaction features a phenyl cation which interacts loosely with both water and dinitrogen (**350**) via pathway (ii) in Scheme 48 A, despite the fact that pathway (i) has a lower Δ*G* ^*^. This is explained by the fact that a phenyl cation **348** would not really exist in aqueous solutions, and the C−N bond cleavage could never evolve to completion without water binding to the developing phenyl cation. The transition state was shown to involve the “out‐of‐plane” attack **352 a** rather than the “in‐plane” attack **352 b** (Scheme 48 B).^113^

Singleton and Ussing have also studied the hydrolysis of arenediazonium cations in water, and do not agree with the results of Glaser, due to there being no consideration of entropic values in Glaser's work, and the fact that it does not agree with kinetic data.^115^ Kinetic isotope effects for ^13^C indicated that there is significant weakening not only of the C_1_−C_2_ bond in the rate‐determining step, but also of the C_2_−C_3_ bonds (see **353**, Scheme 48 C). This is consistent with a structure resembling a distorted aryl cation in the transition state, as C_1_ gains some sp character as a cation. The authors point out that in the transition state, both N_2_ and water are distant from the forming cation, and that the mechanism lies somewhere between S_N_1Ar and S_N_2Ar.

## 12 Reactions of Metal Nucleophiles with Fluorinated Arenes

**Scheme 49:**
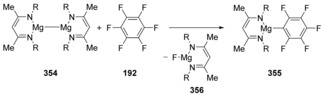
Recent remarkable displacements by magnesium nucleophiles.

The displacement of a fluoride atom from polyfluoroarenes with a magnesium(I) complex was studied experimentally and computationally by Crimmin et al.^116^ The mechanism was found to proceed via a concerted S_N_Ar pathway (Scheme 49). The activation energy found by the DFT method (25.7 kcal mol^−1^) was in good agreement with the experimentally determined activation energy (21.3 kcal mol^−1^). A similar mechanism was found by DFT for the corresponding bimetallic Mg–Zn complex. In this complex the zinc centre acts as the nucleophile. In an earlier study on the Mg–Mg complex,^117^ experimental evidence speaking against single electron pathways was gathered. A S_N_Ar mechanism was proposed and predicted to be concerted by DFT.

In a very recent study with an analogous fluoride‐metal exchange reaction with a corresponding bimetallic Mg–Fe complex, a cS_N_Ar pathway was identified by DFT.^118^ However, an alternative step‐wise S_N_Ar mechanism was found to have a lower overall activation energy. With the Mg–Fe complex, the iron atom acts as the nucleophile.

## 13 An Updated Perspective Emerges on the Prevalence of cSNAr Reactions.

**Scheme 50:**
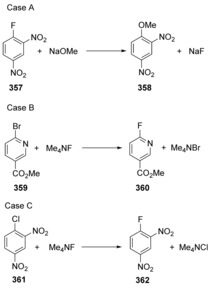
Three reactions studied in depth by Jacobsen et al.

Building on computational and experimental observations, notably from the Ritter group, Jacobsen et al. recently surveyed^14^ S_N_Ar reactions by a combination of experimental and computational methods (Scheme 50). In advance, they based their expectations on the fact that isolated Meisenheimer intermediates can arise when i) substituents on the arene undergoing substitution provide good stabilisation of an intermediate anion, and ii) where the leaving group is relatively poor, so that the intermediate has some kinetic stability. Specifically they initially studied three reactions. Case A satisfies both of the above criteria, Case B features substituents that do not provide such good stabilisation of negative charge, and also boasts an excellent leaving group, bromide, while Case C features substituents that can provide excellent stabilisation while also bearing an excellent leaving group. As such, Case A would likely be a classical S_N_Ar reaction, Case B would likely be concerted and Case C could be borderline between the two mechanistic extremes. Their experimental approach was based on studying kinetic isotope effects in substrates that involve fluoride as a leaving group or as a nucleophile in S_N_Ar reactions. If a kinetic isotope effect is involved in the formation or cleavage of the C−F bond, then this will be reflected in a ^13^C/^12^C isotope effect for that carbon. NMR methods for determining isotope effects were greatly developed by Singleton and Thomas^119^ in ^13^C spectra, but the novel development of Jacobsen et al. is to make use of the NMR sensitivity of the ^19^F nucleus. Studying multiple quantum filtered (MQF) ^19^F{^1^H} spectra allowed clear observation and quantitation of the ^13^C–^19^F satellites to the ^12^C–^19^F peak with very accessible acquisition times for reasonable quantities of substrate (the MQF technique suppresses the appearance of the latter peak).

With the isotope effects measured, the important point was to compare this figure with that calculated using benchmarked computational methods, which also indicate whether an intermediate or a transition state is present. A key indicator of the concerted or stepwise nature of the reaction involving C−F formation or rupture relates to a comparison of this KIE to the maximum computed KIE on the reaction energy surface. Strong bonds in the ground state can lead to loss of more vibrational energy in the TS and therefore to large KIEs.

The largest KIE values arise when the bonding to both nucleophile and leaving group are weak in the TS, that is, in concerted reactions. For example in Case A, a strong C−F bond is broken, leading to large maximum KIE (1.070). In contrast, in Case B, a weak C−Br bond is broken as reflected in the lower maximum KIE (1.045). The measured KIE in both cases was 1.035 but this represents 47 % of the maximum KIE for Case A, but 87 % of the maximum KIE for case B. This translates to a stepwise nature for Case A and a concerted reaction for Case B. They then extended their studies to 120 S_N_Ar reactions with a variety of arene ring types, nucleophiles and leaving groups. Their calculations showed that 99 of the selected substitution reactions (83 %) proceed with concerted mechanisms.

## 14 Summary and Outlook

In 2013, aromatic nucleophilic substitutions were reviewed, and the classical stepwise mechanism was deemed to be the usual mechanism, while concerted nucleophilic substitutions were very rare.^3^ The past six years have certainly built on the undercurrent that existed before 2013 and it is likely that a torrent of concerted examples will appear in the next few years. Investigations have been helped by computational techniques that shed light on the mechanisms. What is clear is that the concerted or stepwise nature of the reactions is strongly influenced by substrate nucleophile and leaving group, but also by the environment, and that some substitutions may present as concerted or stepwise depending on the conditions. We need to be careful about information from Hammett correlations for at least two reasons: i) Hammett *ρ*‐values depend on the temperature at which the experiments are performed and so comparisons need to bear this in mind; ii) if a particular reaction undergoes a transition from stepwise to concerted for a range of substituents on the substrate, this may present as a clear change in *ρ*‐value, but the two pathways could have similar *ρ*‐values, and this could mask the transition. With computational methods, the selection of the method and the basis set clearly influences the outcome of the calculations, and so continued study in this area will be crucial.

With these important changes in perception coming now for nucleophilic aromatic substitution and its implications for Meisenheimer intermediates, it is interesting to see that the counterpart in electrophilic aromatic substitution, that is, concerted electrophilic aromatic substitution, featuring Wheland transition states rather than intermediates, is also beginning to appear.^120^, ^121^, ^122^ We are thus at a time of exciting developments in mechanistic organic chemistry.

## Conflict of interest

The authors declare no conflict of interest.

## Biographical Information

*Simon Rohrbach obtained his Master's Degree in Organic Chemistry at the University of Bern*, *Switzerland*, *before joining the research group of John A. Murphy at the University of Strathclyde*, *UK. He is working on method development and elucidation of complex reaction mechanisms applying both experimental and computational approaches*.

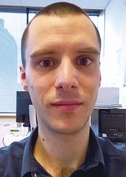

## Biographical Information

*Andrew Smith obtained his MChem in Chemistry from the University of Strathclyde in 2015*, *before starting his PhD work under the supervision of Professor John Murphy at Strathclyde. He is currently studying the mechanistic pathways involved in reducing systems*.

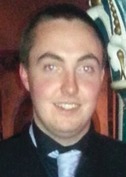

## Biographical Information

*Jia Hao Pang completed his undergraduate studies at Nanyang Technological University (NTU) Singapore in 2016 before beginning his PhD work in the laboratory of Shunsuke Chiba at NTU. He is currently focusing on chemistry of main group metal hydrides for methodology development*.

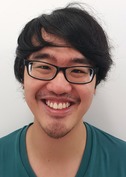

## Biographical Information

*Darren Poole completed his DPhil in 2014 (University of Oxford, Prof Timothy Donohoe). He joined GSK as a synthetic chemist in 2014, and became a Scientific Leader and GSK Associate Fellow in 2018, with a particular interest in applying new technologies to drug discovery*.

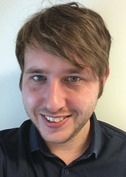

## Biographical Information

*Tell Tuttle earned his PhD in 2004 under the supervision of Prof. Elfi Kraka and Prof. Dieter Cremer at Göteborg University, Sweden. He began his independent career in 2007 and is currently Professor of Theoretical Chemistry at the University of Strathclyde. His research is focused on the use of computational methods for the directed discovery of new reactivities and functional materials*.

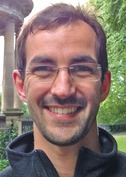

## Biographical Information

*Shunsuke Chiba earned his Ph.D. in 2006 under supervision of Prof. Koichi Narasaka at the University of Tokyo. In 2007, he embarked on his independent career as the faculty of Nanyang Technological University (NTU) Singapore, where he is currently Professor of Chemistry. His research group focuses on methodology development in the area of synthetic chemistry and catalysis*.

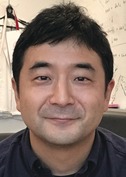

## Biographical Information

*John Murphy was born in Dublin and educated at the University of Dublin (TCD) and the University of Cambridge. After Fellowships at Alberta and Oxford, he was appointed as Lecturer, then Reader, at the University of Nottingham. Since 1995, he has held the Merck–Pauson Professorship at the University of Strathclyde. His interests are in mechanism and synthesis*.

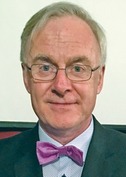

## References

[1] M. B. Smyth , in March's Advanced Organic Chemistry: Reactions, Mechanisms and Structure, 7 ^th^ ed., Wiley, Hoboken, 2013, Chap. 11, pp. 569–641, e-book ISBN: 9781118472217.

[2] J. F. Bunnett , R. E. Zahler , Chem. Rev. 1951, 49, 273–412.

[3] F. Terrier , Modern Nucleophilic Aromatic Substitution, Wiley-VCH, Weinheim, 2013, e-book ISBN: 9783527656141.

[4] K. Błaziak , W. Danikiewicz , M. Mąkosza , J. Am. Chem. Soc. 2016, 138, 7276–7281, and references therein. 27218876 10.1021/jacs.5b13365

[5] H. Takikawa , A. Nishii , T. Sakai , K. Suzuki , Chem. Soc. Rev. 2018, 47, 8030–8056. 30357181 10.1039/c8cs00350e

[6] J. A. Garcia-López , M. F. Greaney , Chem. Soc. Rev. 2016, 45, 6766–6798. 27752670 10.1039/c6cs00220j

[7] R. A. Rossi , A. B. Pierini , A. B. Peñeñory , Chem. Rev. 2003, 103, 71–168. 12517182 10.1021/cr960134o

[8] A. Studer , D. P. Curran , Nat. Chem. 2014, 6, 765–773. 25143210 10.1038/nchem.2031

[9] D. L. Hughes , Org. Prep. Proced. Int. 1993, 25, 607–632.

[10] V. Snieckus , Chem. Rev. 1990, 90, 879–933.

[11] P. Ruiz-Castillo , S. L. Buchwald , Chem. Rev. 2016, 116, 12564–12649. 27689804 10.1021/acs.chemrev.6b00512 PMC5070552

[12] J. F. Hartwig , Acc. Chem. Res. 2012, 45, 864–873. 22075137 10.1021/ar200206a

[13] S. Crespi , S. Protti , M. Fagnoni , J. Org. Chem. 2016, 81, 9612–9619. 27696841 10.1021/acs.joc.6b01619

[14] E. E. Kwan , Y. Zeng , H. A. Besser , E. N. Jacobsen , Nat. Chem. 2018, 10, 917–923. 30013193 10.1038/s41557-018-0079-7 PMC6105541

[15] A. J. J. Lennox , Angew. Chem. Int. Ed. 2018, 57, 14686–14688; 10.1002/anie.201809606 30320484

[16] A. Faust , Ber. Dtsch. Chem. Ges. 1873, 6, 1022–1023.

[17] R. Fittig , E. Mager , Ber. Dtsch. Chem. Ges. 1874, 7, 1175–1180.

[18] P. T. Austen , Ber. Dtsch. Chem. Ges. 1876, 9, 621–623.

[19] J. Meisenheimer , Liebigs Ann. Chem. 1902, 323, 205–246.

[20] G. A. Artamkina , M. P. Egorov , I. P. Beletskaya , Chem. Rev. 1982, 82, 427–459.

[21] K. Ziegler , H. Zeiser , Ber. Dtsch. Chem. Ges. 1930, 63, 1847–1851.

[22] S. D. Robertson , A. R. Kennedy , J. J. Liggat , R. E. Mulvey , Chem. Commun. 2015, 51, 5452–5455. 10.1039/c4cc06421f 25236757

[23] L.-H. Zhang , Z. Tan , Tetrahedron Lett. 2000, 41, 3025–3028.

[24] J. Miller , Aust. J. Chem. 1956, 9, 61–73.

[25] J. Miller , K.-Y. Wan , J. Chem. Soc. 1963, 3492–3495.

[26] C. D. Johnson , The Hammett Equation, Cambridge University Press, Cambridge, 1980.

[27] H. Handel , M. A. Pasquini , J. L. Pierre , Tetrahedron 1980, 36, 3205–3208.

[28] S. E. Fry , N. J. Pienta , J. Am. Chem. Soc. 1985, 107, 6399–6400.

[29] A. Hunter , M. Renfrew , J. A. Taylor , J. M. J. Whitmore , A. Williams , J. Chem. Soc. Perkin Trans. 2 1993, 1703–1704.

[30] A. Hunter , M. Renfrew , D. Rettura , J. A. Taylor , J. M. J. Whitmore , A. Williams , J. Am. Chem. Soc. 1995, 117, 5484–5491.

[31] J. Shakes , C. Raymond , D. Rettura , A. Williams , J. Chem. Soc. Perkin Trans. 2 1996, 1553–1557.

[32] N. R. Cullum , D. Rettura , J. M. J. Whitmore , A. Williams , J. Chem. Soc. Perkin Trans. 2 1996, 1559–1563.

[33] P. Sawunyama , G. W. Bailey , Pest Manage. Sci. 2002, 58, 759–768. 10.1002/ps.522 12192899

[34] M. N. Glukhovtsev , R. D. Bach , S. Laiter , J. Org. Chem. 1997, 62, 4036–4046.

[35] I. Fernández , G. Frenking , E. Uggerud , J. Org. Chem. 2010, 75, 2971–2980. 20353177 10.1021/jo100195w

[36] H. Sun , S. G. DiMagno , Angew. Chem. Int. Ed. 2006, 45, 2720–2725; 10.1002/anie.200504555 16548046

[37] A. Singh , N. Goel , New J. Chem. 2015, 39, 4351–4358.

[38] K. A. Kislyi , A. V. Samet , Y. A. Strelenko , V. V. Semenov , J. Org. Chem. 2008, 73, 2285–2291. 18302412 10.1021/jo702532x

[39] T. Giroldo , L. A. Xavier , J. M. Riveros , Angew. Chem. Int. Ed. 2004, 43, 3588–3590; 10.1002/anie.200454230 15293254

[40] S. Park , S. Lee , Bull. Korean Chem. Soc. 2010, 31, 2571–2574.

[41] K. Tanaka , M. Deguchi , S. Iwata , J. Chem. Res. Synop. 1999, 528–529;

[42] J. Kvíčala , M. Beneš , O. Paleta , V. Král , J. Fluorine Chem. 2010, 131, 1327–1337.

[43] V. M. Vlasov , V. V. Aksenov , P. P. Rodionov , I. V. Beregovaya , L. N. Shchegoleva , Russ. J. Org. Chem. 2002, 38, 115–125.

[44] V. V. Aksenov , V. M. Vlasov , G. G. Yakobson , J. Fluorine Chem. 1982, 20, 439–458.

[45] J. Baker , M. Muir , Can. J. Chem. 2010, 88, 588–597.

[46] M. Liljenberg , T. Brinck , B. Herschend , T. Rein , S. Tomasi , M. Svensson , J. Org. Chem. 2012, 77, 3262–3269. 22384935 10.1021/jo202569n

[47] M. Liljenberg , T. Brinck , T. Rein , M. Svensson , Beilstein J. Org. Chem. 2013, 9, 791–799. 23766792 10.3762/bjoc.9.90 PMC3678587

[48] J. H. Stenlid , T. Brinck , J. Org. Chem. 2017, 82, 3072–3083. 28195731 10.1021/acs.joc.7b00059

[49] I.-H. Um , L.-R. Im , J.-S. Kang , S. S. Bursey , J. M. Dust , J. Org. Chem. 2012, 77, 9738–9746. 23025909 10.1021/jo301862b

[50] J. R. Pliego, Jr. , D. Piló-Veloso , Phys. Chem. Chem. Phys. 2008, 10, 1118–1124. 18270613 10.1039/b716159j

[51] D. R. Silva , J. R. Pliego, Jr. , Struct. Chem. 2019, 30, 75–83.

[52] S. L. C. Moors , B. Brigou , D. Hertsen , B. Pinter , P. Geerlings , V. Van Speybroeck , S. Catak , F. De Proft , J. Org. Chem. 2016, 81, 1635–1644. 26800020 10.1021/acs.joc.5b02794

[53] P. Tang , W. Wang , T. Ritter , J. Am. Chem. Soc. 2011, 133, 11482–11484. 21736304 10.1021/ja2048072 PMC3148096

[54] T. Fujimoto , F. Becker , T. Ritter , Org. Process Res. Dev. 2014, 18, 1041–1044. 25177149 10.1021/op500121w PMC4144717

[55] C. N. Neumann , J. M. Hooker , T. Ritter , Nature 2016, 534, 369–373. 27281221 10.1038/nature17667 PMC4911285

[56] C. N. Neumann , T. Ritter , Acc. Chem. Res. 2017, 50, 2822–2833. 29120599 10.1021/acs.accounts.7b00413

[57] S. D. Schimler , M. A. Cismesia , P. S. Hanley , R. D. J. Froese , M. J. Jansma , D. C. Bland , M. S. Sanford , J. Am. Chem. Soc. 2017, 139, 1452–1455. 28111944 10.1021/jacs.6b12911

[58] A. Kaga , H. Hayashi , H. Hakamata , M. Oi , M. Uchiyama , R. Takita , S. Chiba , Angew. Chem. Int. Ed. 2017, 56, 11807–11811; 10.1002/anie.201705916 28741890

[59] S. Kusumoto , K. Nozaki , Nat. Commun. 2015, 6, 6296. 25704229 10.1038/ncomms7296

[60] J. H. Pang , A. Kaga , S. Chiba , Chem. Commun. 2018, 54, 10324–10327. 10.1039/c8cc05979a 30141796

[61] D. Y. Ong , C. Tejo , K. Xu , H. Hirao , S. Chiba , Angew. Chem. Int. Ed. 2017, 56, 1840–1844; 10.1002/anie.201611495 28071853

[62] For a review, see S. Chiba , K. Ando , K. Narasaka , Synlett 2009, 2549–2564.

[63] J. P. Barham , S. E. Dalton , M. Allison , G. Nocera , A. Young , M. P. John , T. M. McGuire , S. Campos , T. Tuttle , J. A. Murphy , J. Am. Chem. Soc. 2018, 140, 11510–11518. 30119605 10.1021/jacs.8b07632

[64] D. Sadowsky , K. McNeill , C. J. Cramer , Environ. Sci. Technol. 2014, 48, 10904–10911. 25133312 10.1021/es5028822

[65] D. Sadowsky , K. McNeill , C. J. Cramer , Environ. Sci. Technol. 2013, 47, 14194–14203. 24237268 10.1021/es404033y

[66] K. Kikushima , M. Grellier , M. Ohashi , S. Ogoshi , Angew. Chem. Int. Ed. 2017, 56, 16191–16196; 10.1002/anie.201708003 29072350

[67] L. I. Goryunov , J. Grobe , D. Le Van , V. D. Shteingarts , R. Mews , E. Lork , E.-U. Würthwein , Eur. J. Org. Chem. 2010, 1111–1123.

[68] S. I. Zhivetyeva , L. I. Goryunov , I. Y. Bagryanskaya , J. Grobe , V. D. Shteingarts , E.-U. Würthwein , J. Fluorine Chem. 2014, 164, 58–69.

[69] S. Mallick , P. Xu , E.-U. Würthwein , A. Studer , Angew. Chem. Int. Ed. 2019, 58, 283–287; 10.1002/anie.201808646 30387543

[70] K. Kojima , Y. Nagashima , C. Wang , M. Uchiyama , ChemPlusChem, 2019, 84, 277–280. 10.1002/cplu.201900069 31950760

[71] X.-W. Liu , C. Zarate , R. Martin , Angew. Chem. Int. Ed. 2019, 58, 2064–2068; 10.1002/anie.201813294 30575235

[72] A. G. Cairns , H. M. Senn , M. P. Murphy , R. C. Hartley , Chem. Eur. J. 2014, 20, 3742–3751. 24677631 10.1002/chem.201304241 PMC4164275

[73] C. Adouama , M. E. Budén , W. D. Guerra , M. Puiatti , B. Joseph , S. M. Barolo , R. A. Rossi , M. Médebielle , Org. Lett. 2019, 21, 320–324. 30576154 10.1021/acs.orglett.8b03831

[74] E. V. Tretyakov , P. A. Fedyushin , E. V. Panteleeva , D. V. Stass , I. Y. Bagryanskaya , I. V. Beregovaya , A. S. Bogomyakov , J. Org. Chem. 2017, 82, 4179–4185. 28358985 10.1021/acs.joc.7b00144

[75] D. J. Leonard , J. W. Ward , J. Clayden , Nature 2018, 562, 105–109. 30283103 10.1038/s41586-018-0553-9

[76] R. C. Atkinson , D. J. Leonard , J. Maury , D. Castagnolo , N. Volz , J. Clayden , Chem. Commun. 2013, 49, 9734–9736. 10.1039/c3cc46193a 24022183

[77] R. E. Carter , Acta Chem. Scand. 1967, 21, 75–86.

[78] J. E. Hall , J. V. Matlock , J. W. Ward , K. V. Gray , J. Clayden , Angew. Chem. Int. Ed. 2016, 55, 11153–11157; 10.1002/anie.201605714 27440757

[79] H. Abas , J. Mas-Roselló , M. M. Amer , D. J. Durand , R. R. Groleau , N. Fey , J. Clayden , Angew. Chem. Int. Ed. 2019, 58, 2418–2422; 10.1002/anie.201813984 30600901

[80] J. Clayden , U. Hennecke , Org. Lett. 2008, 10, 3567–3570. 18642922 10.1021/ol801332n

[81] R. Costil , Q. Lefebvre , J. Clayden , Angew. Chem. Int. Ed. 2017, 56, 14602–14606; 10.1002/anie.201708991 28967697

[82] J. Lefranc , A. M. Fournier , G. Mingat , S. Herbert , T. Marcelli , J. Clayden , J. Am. Chem. Soc. 2012, 134, 7286–7289. 22480365 10.1021/ja301591m

[83] R. Costil , H. J. A. Dale , N. Fey , G. Whitcombe , J. V. Matlock , J. Clayden , Angew. Chem. Int. Ed. 2017, 56, 12533–12537; 10.1002/anie.201706341 28817222

[84] T. J. Snape , Chem. Soc. Rev. 2008, 37, 2452–2458. 18949118 10.1039/b808960d

[85] F. Nawaz , K. Mohanan , L. Charles , M. Rajzmann , D. Bonne , O. Chuzel , J. Rodriguez , Y. Coquerel , Chem. Eur. J. 2013, 19, 17578–17583. 24318271 10.1002/chem.201303359

[86] L. Legnani , A. Porta , P. Caramella , L. Toma , G. Zanoni , G. Vidari , J. Org. Chem. 2015, 80, 3092–3100. 25685875 10.1021/acs.joc.5b00008

[87] W. E. Truce , E. M. Kreider , W. W. Brand in The Smiles and Related Rearrangements of Aromatic Systems, Wiley, Hoboken, 1970.

[88] N. Chéron , D. Jacquemin , P. Fleurat-Lessard , Phys. Chem. Chem. Phys. 2012, 14, 7170–7175. 22491187 10.1039/c2cp40438a

[89] J. Reyes-González , R. M. Gómez , F. Cortés-Guzman , J. Phys. Org. Chem. 2012, 25, 230–238.

[90] B. Yang , X. Tan , R. Guo , S. Chen , Z. Zhang , X. Chu , C. Xie , D. Zhang , C. Ma , J. Org. Chem. 2014, 79, 8040–8048. 25101862 10.1021/jo5011729

[91] Y. Zhao , Y. Wu , J. Jia , D. Zhang , C. Ma , J. Org. Chem. 2012, 77, 8501–8506. 22950354 10.1021/jo3014287

[92] H. Sun , J. Li , D. Zhang , C. Ma , C. Liu , J. Phys. Org. Chem. 2008, 21, 215–218.

[93] A. Schönberg , L. von Varga , Ber. Dtsch. Chem. Ges. 1930, 63, 178–180.

[94] H. R. Al-Kazimi , D. S. Tarbell , D. Plant , J. Am. Chem. Soc. 1955, 77, 2479–2482.

[95] G. C. Lloyd-Jones , J. D. Moseley , J. S. Renny , Synthesis 2008, 661–689.

[96] H. M. Relles , G. Pizzolato , J. Org. Chem. 1968, 33, 2249–2253.

[97] K. Miyazaki , Tetrahedron Lett. 1968, 9, 2793–2798.

[98] V. Albrow , K. Biswas , A. Crane , N. Chaplin , T. Easun , S. Gladiali , B. Lygo , S. Woodward , Tetrahedron: Asymmetry 2003, 14, 2813–2819.

[99] H. Jacobsen , J. P. Donahue , Can. J. Chem. 2006, 84, 1567–1574.

[100] A. J. Perkowski , C. L. Cruz , D. A. Nicewicz , J. Am. Chem. Soc. 2015, 137, 15684–15687. 26645387 10.1021/jacs.5b11800

[101] S. Chiniforoush , C. J. Cramer , J. Org. Chem. 2019, 84, 2148–2157. 30672704 10.1021/acs.joc.8b03132

[102] S. K. Pedersen , A. Ulfkjaer , M. N. Newman , S. Yogarasa , A. U. Petersen , T. I. Sølling , M. Pittelkow , J. Org. Chem. 2018, 83, 12000–12006. 30160966 10.1021/acs.joc.8b01800

[103] T. Broese , A. F. Roesel , A. Prudlik , R. Francke , Org. Lett. 2018, 20, 7483–7487. 30489089 10.1021/acs.orglett.8b03257

[104] Y. Masuya , Y. Kawashima , T. Kodama , N. Chatani , M. Tobisu , Synlett 2019, 10.1055/s-0037-1611974.

[105] N. H. Park , G. dos P. Gomes , M. Fevre , G. O. Jones , I. V. Alabugin , J. L. Hedrick , Nat. Commun. 2017, 8, 13.28408739

[106] K. Calfumán , S. Gallardo-Fuentes , R. Contreras , R. A. Tapia , P. R. Campodónico , New J. Chem. 2017, 41, 12671–12677.

[107] Y.-J. Zheng , T. C. Bruice , J. Am. Chem. Soc. 1997, 119, 3868–3877.

[108] E. Stridfeldt , E. Lindstedt , M. Reitti , J. Bild , P.-O. Norrby , B. Olofsson , Chem. Eur. J. 2017, 23, 13249–13258. 28792102 10.1002/chem.201703057 PMC5639379

[109] A. H. Sandtorv , D. R. Stuart , Angew. Chem. Int. Ed. 2016, 55, 15812–15815; 10.1002/anie.201610086 27862773

[110] N. Purkait , G. Kervefors , E. Linde , B. Olofsson , Angew. Chem. Int. Ed. 2018, 57, 11427–11431; 10.1002/anie.201807001 PMC6120470 29956877

[111] Y. Masumoto , K. Miyamoto , T. Iuchi , M. Ochiai , K. Hirano , T. Saito , C. Wang , M. Uchiyama , J. Org. Chem. 2018, 83, 289–295. 29183123 10.1021/acs.joc.7b02701

[112] E. G. Bakalbassis , S. Spyroudis , E. Tsiotra , J. Org. Chem. 2006, 71, 7060–7062. 16930064 10.1021/jo0610964

[113] Z. Wu , R. Glaser , J. Am. Chem. Soc. 2004, 126, 10632–10639. 15327321 10.1021/ja047620a

[114] For complementary work on diazonium salts, see: I. M. Cuccovia , M. A. da Silva , H. M. C. Ferraz , J. R. Pliego, Jr. , J. M. Riveros , H. Chaimovich , J. Chem. Soc. Perkin Trans. 2 2000, 1896–1907.

[115] B. R. Ussing , D. A. Singleton , J. Am. Chem. Soc. 2005, 127, 2888–2899. 15740124 10.1021/ja043918p

[116] C. Bakewell , B. J. Ward , A. J. P. White , M. R. Crimmin , Chem. Sci. 2018, 9, 2348–2356. 29719707 10.1039/c7sc05059c PMC5897846

[117] C. Bakewell , A. J. P. White , M. R. Crimmin , J. Am. Chem. Soc. 2016, 138, 12763–12766. 27636244 10.1021/jacs.6b08104 PMC5135227

[118] M. Garçon , C. Bakewell , A. J. P. White , M. R. Crimmin , Chem. Commun. 2019, 55, 1805–1808. 10.1039/c8cc09701a 30667423

[119] D. A. Singleton , A. A. Thomas , J. Am. Chem. Soc. 1995, 117, 9357–9358.

[120] G. J. P. Perry , J. M. Quibell , A. Panigrahi , I. Larrosa , J. Am. Chem. Soc. 2017, 139, 11527–11536. 28735532 10.1021/jacs.7b05155 PMC5662929

[121] J. M. Quibell , G. J. P. Perry , D. M. Cannas , I. Larrosa , Chem. Sci. 2018, 9, 3860–3865. 29780518 10.1039/c8sc01016a PMC5935059

[122] B. U. W. Maes , S. Verbeeck , T. Verheist , A. Ekomié , N. von Wolff , G. Lefēvre , E. A. Mitchell , A. Jutand , Chem. Eur. J. 2015, 21, 7858–7865. 25858175 10.1002/chem.201406210

